# Efficacy of irreversible electroporation combined with immunotherapy versus irreversible electroporation alone in locally advanced pancreatic cancer: a propensity score-matched retrospective study

**DOI:** 10.3389/fimmu.2025.1620988

**Published:** 2025-07-08

**Authors:** Pu Xi, Peng Sun, Miao Chen, Zehui Yao, Qi Zhu, Shengping Li, Chaobin He

**Affiliations:** ^1^ Department of Pancreatobiliary Surgery, State Key Laboratory of Oncology in South China, Guangdong Provincial Clinical Research Center for Cancer, Collaborative Innovation Center for Cancer Medicine, Sun Yat-sen University Cancer Center, Guangzhou, Guangdong, China; ^2^ Department of Anesthesiology, State Key Laboratory of Oncology in South China, Guangdong Provincial Clinical Research Center for Cancer, Collaborative Innovation Center for Cancer Medicine, Sun Yat-sen University Cancer Center, Guangzhou, Guangdong, China; ^3^ Department of Nuclear Medicine, The Central Hospital of Wuhan, Tongji Medical College, Huazhong University of Science and Technology, Wuhan, China

**Keywords:** locally advanced pancreatic cancer, irreversible electroporation, immunotherapy, efficacy, prognosis

## Abstract

**Background:**

Irreversible electroporation (IRE) has shown promise in improving survival outcomes and activating the immune response in patients with locally advanced pancreatic cancer (LAPC). Given these immune-enhancing effects, we hypothesized that combining IRE with immune checkpoint inhibitors may further improve treatment outcomes. This study aimed to evaluate the efficacy and safety of IRE combined with anti-PD-1 immunotherapy versus IRE alone in patients with LAPC.

**Methods:**

In this retrospective study, LAPC patients treated either with IRE plus toripalimab (240 mg administered 7 days post-IRE) or with IRE alone were included. Propensity score matching (PSM) analyses were employed for analysis. Clinical outcomes including overall survival (OS), progression-free survival (PFS), and treatment-related adverse events were analyzed and compared between the groups.

**Results:**

A total of 108 patients from August 2015 and Match 2024 from SYSUCC cohort were identified with 76 undergoing IRE and 32 undergoing IRE and toripalimab in this study. After PSM, 96 patients consisting of 64 and 32 patients in the IRE and combination groups were enrolled. Clinical factors were all balanced between two groups. Patients receiving IRE combined with toripalimab showed significantly improved OS (35.03 months; 95% CI: 30.94-39.13 vs. 15.87 months; 95% CI: 8.99-22.74; P=0.014) and PFS (14.33months; 95% CI: 11.19-17.47 vs. 7.47 months; 95% CI: 3.86-11.08; P=0.022) compared to those receiving IRE alone. No treatment-related mortality was reported in either group and no statistically significant differences were observed in terms of complications and adverse events between two groups (all P>0.05).

**Conclusions:**

The combination of IRE and anti-PD-1 immunotherapy was associated with improved survival outcomes and acceptable safety profiles compared to IRE alone in patients with LAPC. Further investigation through prospective trials is warranted.

## Introduction

Pancreatic ductal adenocarcinoma (PDAC) is a highly aggressive gastrointestinal malignancy characterized by rising incidence and a substantial impact on cancer-related mortality globally (1). Approximately 40% of PDAC cases present as locally advanced pancreatic cancer (LAPC), defined by the involvement of major vascular structures, resulting in unresectable yet non-metastatic disease. Despite current treatment modalities, LAPC prognosis remains poor, with a median survival around 12 months (2). Identifying an optimal therapeutic strategy for LAPC remains a significant clinical challenge. Chemotherapy has expanded treatment options, including the potential for tumor downstaging and subsequent surgical resection. Although some LAPC patients benefit from extended surgical resection following chemotherapy, the rates of successful conversion surgery vary widely (0%–43%), influenced by factors such as chemotherapy regimens, tumor heterogeneity, and surgical techniques (3). Additionally, extended surgeries are associated with relatively high postoperative complication rates, potentially diminishing survival benefits (4). Given that mortality in LAPC patients is primarily driven by local tumor progression rather than distant metastasis, local ablative therapies represent a valuable therapeutic avenue (5).

Local ablative therapies have emerged as important adjunctive treatments for LAPC. Nevertheless, conventional thermal ablative methods, such as radiofrequency ablation (RFA) and microwave ablation, are restricted due to potential thermal injury to nearby organs and vessels (6, 7). Irreversible electroporation (IRE), a non-thermal ablative technique, induces apoptosis through permeabilization of the tumor cell membranes by applying short, high-voltage electrical pulses (8). Notably, IRE’s independence from the heat sink effect makes it particularly suitable for LAPC compared to thermal methods. Furthermore, the vascular preservation associated with IRE facilitates the transport of immune cells and molecules, enhancing its immunological responsiveness relative to thermal ablation techniques.

Beyond direct tumor cell apoptosis, IRE has been demonstrated to remodel the tumor microenvironment (TME) and stimulate immune responses (9, 10). Prior research indicates that IRE reduces immune suppression and enhances T-cell activation, suggesting its potential to augment immunotherapy efficacy in PDAC (11, 12).

In recent years, immune checkpoint inhibitors (ICIs) have significantly advanced treatment outcomes in cancers such as melanoma, lung, and liver cancers (13–15). However, the therapeutic benefits of ICIs in PDAC remain limited, potentially due to low programmed cell death protein 1 (PD-1) expression, low mutational burden, limited T-cell infiltration, and increased regulatory T-cell (Treg) accumulation (16, 17). Efforts have thus focused on combination therapies aimed at modifying the immunosuppressive TME to improve ICI responsiveness.

IRE has demonstrated the ability to induce immunogenic cell death (ICD), enhancing effector CD8^+^ T-cell infiltration (9). Moreover, it facilitates antigen presentation by encouraging dendritic cell maturation and promoting M1 macrophage polarization (10, 18). These properties position IRE as a promising adjunct therapy capable of transforming the immunologically “cold” TME into a “hot” environment, thereby improving ICI responsiveness. Indeed, preclinical studies combining IRE and anti-PD-1 therapy have shown increased selective infiltration of CD8^+^ T cells and significantly prolonged survival in Kras-induced pancreatic cancer (KPC) models (19). Additionally, previous studies based on small cohorts had shown that the combination of IRE and anti-PD-1 therapy provided encouraging survival results for LAPC (20, 21). Despite these promising results, the clinical benefit of combining IRE with anti-PD-1 therapy based on relatively large cohorts with long time follow-up in LAPC remains necessary. Therefore, this study was designed to evaluate the clinical outcomes and survival of LAPC patients undergoing combined IRE and anti-PD-1 therapy, aiming to validate the potential therapeutic benefits observed in preclinical settings.

## Methods

### Patients

This retrospective study adhered to the ethical guidelines established by the 1964 Helsinki Declaration and received approval from the Institutional Review Board of Sun Yat-sen University Cancer Center (SYSUCC). Informed consent was obtained from all participants before initiating treatment. Eligible patients were identified through electronic medical records based on these inclusion criteria: (1) histologically confirmed pancreatic adenocarcinoma with radiologically confirmed locally advanced pancreatic cancer (LAPC), defined according to the seventh edition of the AJCC staging system, which includes arterial involvement of the celiac axis or superior mesenteric artery, or unreconstructable involvement of the superior mesenteric or portal vein without metastatic disease confirmed by abdominal and thoracic computed tomography (CT) (22); (2) Eastern Cooperative Oncology Group (ECOG) performance status score of 0–2. Patients who were lost to follow-up or had incomplete information of follow-up were excluded from this study.

### Treatment procedure

The procedure for IRE followed previously reported methods (23). Two to six probes were positioned around the tumor based on its dimensions to establish an electric field, resulting in nanoscale pores in tumor cell membranes. The generator software optimized probe placement based on ultrasound data, specifying appropriate voltage and pulse duration. Standard settings included an initial voltage of 1500 V/cm with 90 pulses at pulse durations of 70–90 ms.

Chemotherapy is the standard treatment for LAPC according to the guideline of National Comprehensive Cancer Network (NCCN) and it was adopted for all patients in this study. Patients received induction and adjuvant chemotherapy with the FOLFIRINOX (a combination of folinic acid, 5-fluorouracil, irinotecan, and oxaliplatin), gemcitabine with nab-paclitaxel (AG) or S-1 (Tegafur, Gimeracil, and Oteracil Potassium Capsules) regimen for 4 months as previously described (24, 25). On the base of the standard care (chemotherapy), the immunotherapy was recommended for part of patients according to doctors’ experience. Written informed consent of immunotherapy was obtained from these patients. In these patients, anti-PD-1 therapy (Toripalimab 240 mg) was initiated one week post-IRE and administered every three weeks thereafter. Patients who had received IRE treatment were included in the IRE group. Those who had received IRE combined with anti-PD-1 therapy were included in combination group.

### Data collection

Patient data, including clinical and radiological information, were retrospectively extracted from medical records. Collected data included demographics (age, gender), tumor characteristics (size, grade, location), laboratory parameters (white blood cell count, platelet count, alanine transaminase, aspartate aminotransferase, alkaline phosphatase, gamma-glutamyl transpeptidase, albumin, total bilirubin, indirect bilirubin, C-reactive protein, hepatitis B surface antigen, carcinoembryonic antigen, carbohydrate antigen 19-9), and chemotherapy regimens. Primary endpoints of the study were overall survival (OS) and progression-free survival (PFS), calculated from the date of diagnosis until death from any cause, disease progression, or last follow-up. Follow-up concluded on Match 30, 2025.

### Statistical analysis

Propensity score matching (PSM) analysis at a ratio of 1:2 was used to minimize selection bias and balance variables. Propensity scores for all patients were estimated by a logistic regression model using the following characteristics as covariates: age, gender, tumor size, imaging LN metastasis, response to neoadjuvant chemotherapy, adjuvant chemotherapy, CA19–9 and CA12-5. A one-to-one nearest-neighbor matching algorithm with an optimal of 0.2 without replacement was used. Categorical variables were compared using the chi-square test or Fisher’s exact test and reported as frequencies and percentages. Variables significantly correlated with OS in univariate analysis were entered into multivariate Cox regression to identify independent predictors, expressed with 95% confidence intervals (CI). OS and PFS curves were analyzed using the Kaplan-Meier method, and differences between the groups were identified using the log-rank test.

Analyses for survival curves were performed using MedCalc software version 11.4.2.0 (MedCalc, Ostend, Belgium). All statistical analyses were conducted with R software version 3.4.2 (The R Foundation for Statistical Computing, Vienna, Austria). A two-tailed P-value of <0.05 was deemed statistically significant.

## Results

### Patient characteristics

**Figure 1:**
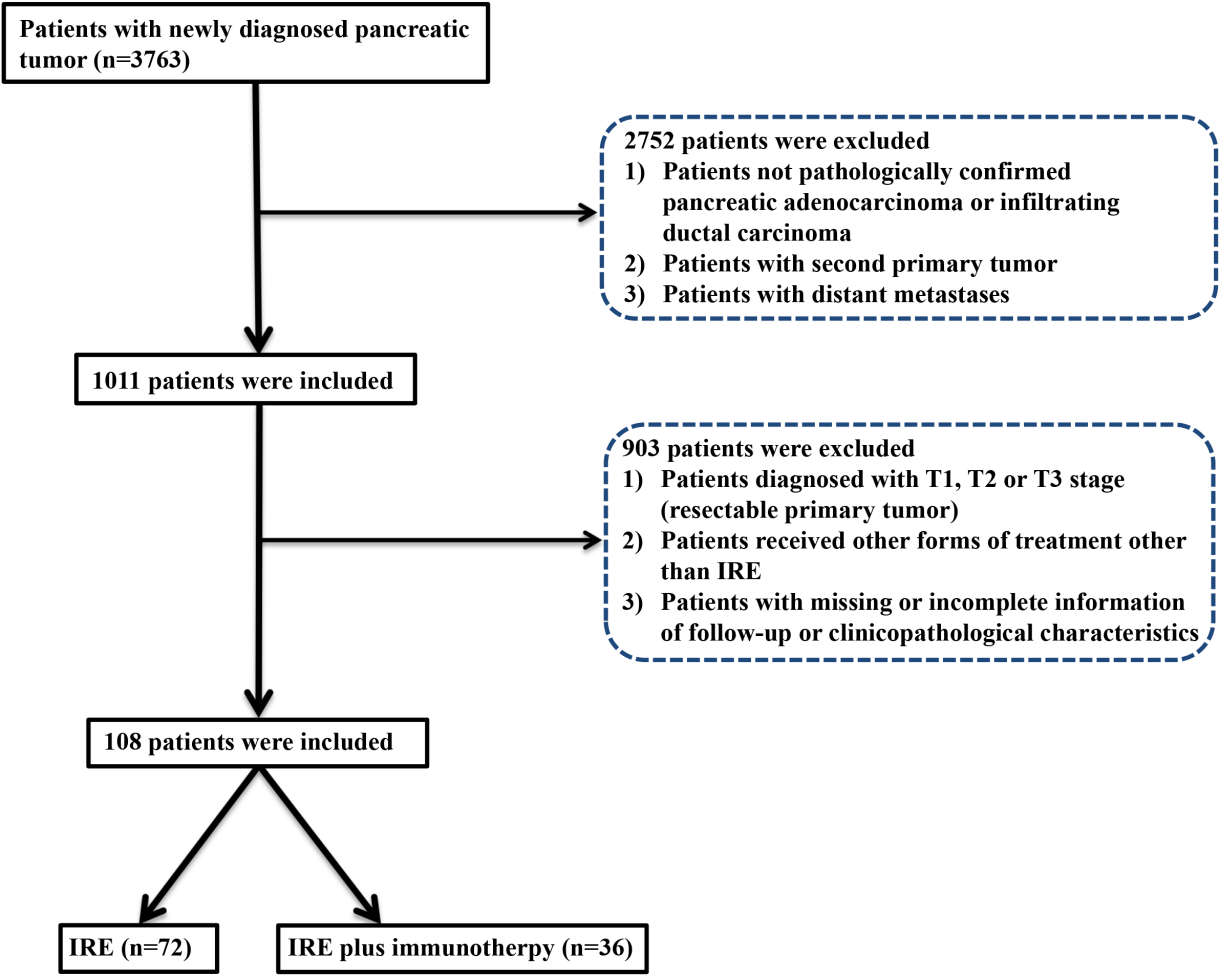
Flowchart of the included patients.

From August 2015 to Match 2023, a total of 108 eligible patients were enrolled: 32 received combined therapy with irreversible electroporation (IRE) and toripalimab (anti-PD-1 therapy), and 76 received IRE alone. A total of 21 patients with missing or incomplete information of follow-up or clinicopathological characteristics were excluded in this study (**Figure 1**). For patients included in this study, chemotherapy for a total of four months were adopted before IRE. Baseline clinical and pathological characteristics were balanced between groups. Nearly balanced distribution of gender was observed in the whole group. The median age was 57.5 years (range, 19–87 years), specifically 57.8 years (range, 40–76 years) for the combined treatment group and 57.3 years (range, 19–87 years) for the IRE-alone group. Tumors shared similar characteristics between two groups, including tumor size, grade, site and vascular invasion. Additionally, adjuvant therapy, such as radiotherapy, targeted therapy and chemotherapy were also similar between these two groups (**Table 1**). PSM was further applied to minimize the selection bias at a caliper score of 0.1 and match ratio of 2:1. After PSM, there were 32 and 64 patients in the combined treatment therapy and IRE group, respectively. No differences in the baseline characteristics after PSM across groups were observed (**Supplementary Table S1**).

**Table 1:** Clinicopathological characteristics of patients with LAPC stratified by treatment.

Variable		Treatment		N	P	Variable		Treatment		N	P
		IRE	IRE+PD1	108				IRE	IRE+PD1	108	
Age (years)	≤ 60	46	18	64	0.830	Tumor grade	Well	6	2	8	0.608
	> 60	30	14	44			Moderate	42	21	63	
Gender	Male	42	11	53	0.059		Poor	28	9	37	
	Female	34	21	55		Tumor size (cm)	≤ 2	1	1	2	0.490
WBC (*10^9^)	≤ 10	69	30	99	0.899		2~4	48	23	71	
	> 10	7	2	9			> 4	27	8	35	
HGB (g/L)	≤ 125	26	8	34	0.375	Tumor site	Head	36	14	50	0.833
	> 125	50	24	74			Body/tail	40	18	58	
PLT (*10^9^)	≤ 350	71	27	98	0.158	Imaging LN metastasis	Absence	34	10	44	0.207
	> 350	5	5	10			Presence	42	22	64	
ALT (U/L)	≤ 50	63	26	89	0.838	Vascular invasion type	Vein	64	29	93	0.545
	> 50	13	6	19			Artery	12	3	15	
AST (U/L)	≤ 40	63	30	93	0.222	Response to NAC	PR	19	15	34	0.081
	> 40	13	2	15			SD	48	14	62	
ALP (U/L)	≤ 125	46	20	66	0.848		PD	9	3	12	
	> 125	30	12	42		Tageted therapy	Absence	71	26	97	0.080
GGT (U/L)	≤ 60	44	23	67	0.198		Presence	5	6	11	
	> 60	32	9	41		HBsAg	Absence	72	30	102	0.838
ALB (g/L)	> 40	16	6	22	0.992		Presence	4	2	6	
	≤ 40	60	26	86		Adjuvant chemotherapy	S-1	53	18	71	0.392
TBIL (umol/L)	≤ 20.5	60	27	87	0.603		AG	9	6	15	
	> 20.5	16	5	21			FOLFIRINOX	14	8	22	
IBIL (umol/L)	≤ 15	67	30	97	0.501	Radiotherapy	Absence	62	22	84	0.204
	> 15	9	2	11			Presence	14	10	24	
CRP (ng/L)	≤3	52	22	74	0.973	CA19-9 (U/ml)	≤ 35	26	12	38	0.826
	> 3	24	10	34			>35	50	20	70	
CEA (ng/ml)	≤ 5	47	26	73	0.071	CA125	≤ 35	58	30	88	0.055
	> 5	29	6	35			>35	18	2	20	

WBC, white blood cell; PLT, platelet; ALT, alanine transaminase; AST, aspartate aminotransferase; ALP, alkaline phosphatase; GGT, glutamyl transpeptidase; ALB, albumin; TBIL, total bilirubin; IBIL, indirect bilirubin; CRP, C-reactive protein; HBsAg, hepatitis B surface antigen; CEA, carcinoembryonic antigen; CA19-9, carbohydrate antigen 19-9; AG, Abraxane-GEM; FOLFIRINOX, leucovorin, fluorouracil, irinotecan, and oxalipatin; LN, lymph node; NAC, neoadjuvant chemotherapy.

### Survival analysis

**Figure 2:**
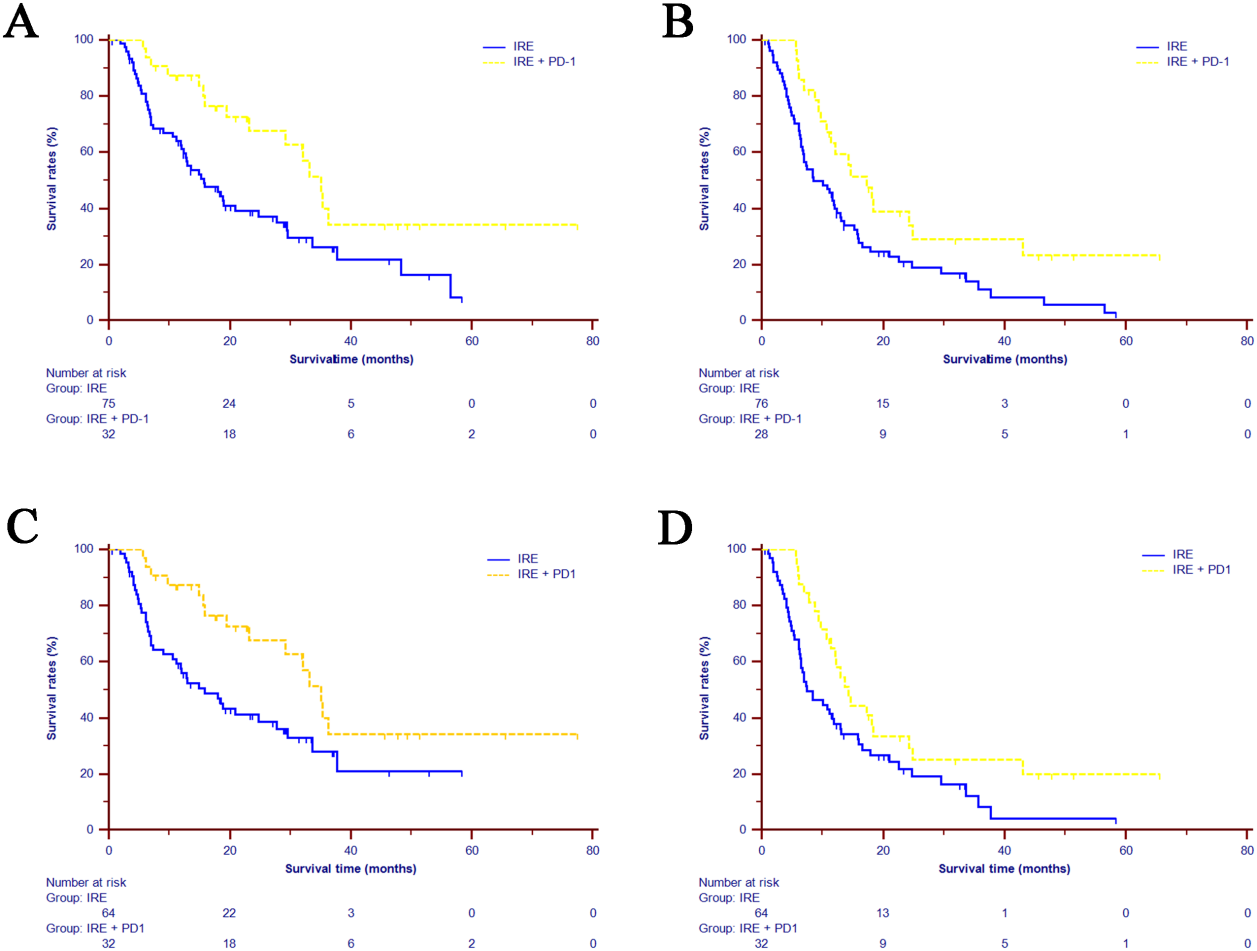
Kaplan-Meier estimates of survival for the LAPC patients underwent IRE and combined treatment (IRE+PD-1). **(A)** OS comparison in the whole cohort. **(B)** PFS comparison in the whole cohort. **(C)** OS comparison in the matched cohort. **(D)** PFS comparison in the matched cohort. LAPC=locally advanced pancreatic cancer; IRE=irreversible electroporation; OS=overall survival; PFS=progression-free survival.

With a median follow-up of 26.5 months and the longest follow-up of 69.2 months, the median overall survival (OS) for the entire cohort was 20.93 months (95% CI: 11.38-30.49 months), and median progression-free survival (PFS) was 11.70 months (95% CI: 9.68–13.72 months). The median survival time (MST) of OS was significantly longer in the combined treatment group (35.03 months, 95% CI: 30.94–39.13 months) compared to the IRE-alone group (15.77 months, 95% CI: 10.23–21.31 months). The 1-, 2-, and 3-year OS rates were 83.7%, 67.6%, and 34.2%, respectively, in the combined group versus 63.1%, 38.1%, and 27.4% in the IRE-alone group (P=0.008, **Figure 2A**). The MST of PFS was also significantly improved in the combined group (14.33 months, 95% CI: 11.19-17.43 months) compared to the IRE-alone group (8.53 months, 95% CI: 4.05–13.02 months). One- and two-year PFS rates were 61.3% and 29.3% for the combined group versus 41.2% and 18.8% for the IRE-alone group (P=0.024, **Figure 2B**). After PSM, significantly higher OS and PFS rates were also observed in the combined treatment group, compared with IRE group [OS: MST, 35.03 months (95% CI: 11.38-30.49 months) vs. 15.87 months (95% CI: 8.99-22.74 months), P=0.014, **Figure 2C**; PFS: MST, 14.33 months (95% CI: 11.19-17.47 months) vs. 7.47 months (95% CI: 3.86-11.08 months), P=0.022, **Figure 2D**].

### Prognostic factors for OS and PFS

To identify risk factors for OS and PFS, all clinical and pathological factors were included and analyzed using Cox regression analysis. The results showed that anti-PD1 therapy, age, vascular invasion type, tumor response after neoadjuvant chemotherapy, AST and tumor grade were associated with OS. Further multivariate analysis revealed that age older than 60 years old (HR=1.816, 95% CI 1.056–3.124, P=0.031) predicted poorer OS compared to those with younger ages. Additionally, tumor of poor differentiation (HR=4.735, 95% CI 1.312-17.088, P=0.018) and arterial invasion (HR=2.324, 95% CI 1.166-4.633, P=0.017) were associated with worse survival, while anti-PD1 therapy (HR=0.497, 95% CI 0.269–0.917, P=0.025) was likely to prolong OS (**Supplementary Table S2**). After PSM, anti-PD1 therapy (HR=0.504, 95% CI 0.274–0.925, P=0.027) was significant prognostic factors for OS (**Table 2**). For PFS, multivariate analysis indicated that adjuvant chemotherapy regimen (AG vs. S-1, HR=2.216, 95% CI 1.190–4.129, P=0.012) and anti-PD1 therapy (HR=0.537, 95% CI 0.318–0.909, P=0.020) were significant predictive factors for PFS in patients with LAPC (**Supplementary Table S3**). In the matched cohorts after PSM, these two factors were also identified as prognostic factors for PFS (**Table 3**).

**Table 2:** Independent prognostic factors for OS in matched cohort.

Characteristics		OS					
		Univariate analysis			Multivariate analysis		
		HR	95%CI	P	HR	95%	P
Age (years)	≤ 60	reference		0.016	Reference		0.094
	> 60	1.917	1.127-3.260		1.615	0.921-2.832	
Gender	Male	reference		0.2212	Reference		
	Female	0.712	0.417-1.214				
WBC (*10^9^)	≤ 10	reference		0.956	Reference		
	> 10	1.026	0.408-2.579				
HGB (g/L)	≤ 120	reference		0.303	Reference		
	> 120	0.748	0.431-1.299				
PLT (*10^9^)	≤ 300	reference		0.748	Reference		
	> 300	1.139	0.515-2.519				
ALT (U/L)	≤ 50	reference		0.520	Reference		
	> 50	1.255	0.628-2.505				
AST (U/L)	≤ 40	reference		0.150	Reference		
	> 40	1.697	0.826-3.488				
ALP (U/L)	≤ 125	Reference		0.389	Reference		
	> 125	0.789	0.459-1.355				
GGT (U/L)	≤ 60	Reference		0.569	Reference		
	> 60	1.171	0.681-2.013				
ALB (g/L)	> 40	Reference		0.195	Reference		
	≤ 40	0.654	0.344-1.243				
TBIL (umol/L)	≤ 20.5	Reference		0.576	Reference		
	> 20.5	0.828	0.427-1.606				
IBIL(umol/L)	≤ 15	Reference		0.876	Reference		
	> 15	0.939	0.424-2.078				
CRP (ng/L)	≤3	Reference		0.208	Reference		
	> 3	1.424	0.821-2.469				
CEA (ng/mL)	≤ 5	Reference		0.638	Reference		
	> 5	0.866	0.475-1.578				
CA19-9 (U/ml)	≤ 35	Reference		0.096	Reference		0.173
	>35	1.697	0.911-3.162		1.552	0.824-2.922	
CA125	≤ 35	Reference		0.368	Reference		
	>35	1.346	0.705-2.569				
Tumor size	≤ 2	Reference		0.335	Reference		
	2~4	2.445	0.332-17.994	0.380			
	> 4	3.396	0.444-25.963	0.239			
Tumor site	Head	Reference		0.594	Reference		
	Body/tail	0.864	0.506-1.477				
Tumor grade	Well	Reference		0.143	Reference		
	Moderate	1.622	0.494-5.322	0.425			
	Poor	2.575	0.765-8.666	0.127			
Imaging LN metastasis	Absence	Reference		0.258	Reference		
	Presence	0.733	0.428-1.256				
Vascular invasion type	Vein	Reference		0.067	Reference		0.244
	Artery	1.901	0.956-3.781		1.525	0.750-3.103	
Neoadjuvant radiotherapy	Absence	Reference		0.248	Reference		
	Presence	0.689	0.367-1.295				
Response to NCP	PR	Reference		0.036	Reference		0.125
	SD	2.129	1.161-3.903	0.015	1.744	0.918-3.314	0.089
	PD	1.147	0.417-3.159	0.791	0.862	0.308-2.147	0.778
Adjuvant chemotherapy	S-1	Reference		0.264	Reference		
	AG	1.287	0.630-2.627	0.489			
	FOLFIRINOX	0.650	0.335-1.262	0.203			
Tageted therapy	Absence	Reference		0.591	Reference		
	Presence	0.792	0.338-1.855				
HBsAg	Absence	Reference		0.380	Reference		
	Presence	0.530	0.129-2.184				
PD1	Absence			0.017	Reference		0.027
	Presence	0.482	0.265-0.876		0.504	0.274-0.925	

OS, overall survival; HR, hazard ratio; CI, confidence interval; NI, not include, other abbreviations as in **Table 1**.

**Table 3:** Independent prognostic factors for PFS in matched cohort.

Characteristics		OS					
		Univariate analysis			Multivariate analysis		
		HR	95%CI	P	HR	95%	P
Age (years)	≤ 60	reference		0.301	Reference		
	> 60	1.274	0.805-2.016				
Gender	Male	reference		0.392	Reference		
	Female	1.227	0.768-1.961				
WBC (*10^9^)	≤ 10	reference		0.618	Reference		
	> 10	0.808	0.349-1.869				
HGB (g/L)	≤ 120	reference		0.882	Reference		
	> 120	1.038	0.637-1.690				
PLT (*10^9^)	≤ 300	reference		0.562	Reference		
	> 300	0.794	0.364-1.731				
ALT (U/L)	≤ 50	reference		0.739	Reference		
	> 50	0.896	0.469-1.710				
AST (U/L)	≤ 40	reference		0.822	Reference		
	> 40	1.084	0.538-2.185				
ALP (U/L)	≤ 125	Reference		0.173	Reference		
	> 125	0.721	0.450-1.154				
GGT (U/L)	≤ 60	Reference		0.839	Reference		
	> 60	1.050	0.657-1.679				
ALB (g/L)	> 40	Reference		0.494	Reference		
	≤ 40	0.815	0.454-1.464				
TBIL (umol/L)	≤ 20.5	Reference		0.193	Reference		
	> 20.5	0.678	0.377-1.217				
IBIL(umol/L)	≤ 15	Reference		0.553	Reference		
	> 15	1.236	0.614-2.487				
CRP (ng/L)	≤3	Reference		0.017	Reference		0.196
	> 3	1.798	0.113-2.905		1.414	0.836-2.393	
CEA (ng/mL)	≤ 5	Reference		0.865	Reference		
	> 5	1.045	0.629-1.736				
CA19-9 (U/ml)	≤ 35	Reference		0.018	Reference		0.199
	>35	1.907	1.119-3.247		1.466	0.817-2.629	
CA125	≤ 35	Reference		0.536	Reference		
	>35	1.206	0.667-2.179				
Tumor size	≤ 2	Reference		0.159	Reference		
	2~4	3.411	0.468-24.836	0.226			
	> 4	4.856	0.649-36.326	0.124			
Tumor site	Head	Reference		0.928	Reference		
	Body/tail	1.022	0.641-1.629				
Tumor grade	Well	Reference		0.677	Reference		
	Moderate	0.901	0.405-2.006	0.798			
	Poor	1.132	0.484-2.645	0.775			
Imaging LN metastasis	Absence	Reference		0.082	Reference		0.203
	Presence	0.662	0.416-1.053		0.725	0.442-1.190	
Vascular invasion type	Vein	Reference		0.585	Reference		
	Artery	1.197	0.628-2.282				
Neoadjuvant radiotherapy	Absence	Reference		0.950	Reference		
	Presence	1.017	0.612-1.687				
Response to NCP	PR	Reference		0.459	Reference		
	SD	1.253	0.766-2.050	0.369			
	PD	0.814	0.353-1.876	0.629			
Adjuvant chemotherapy	S-1	Reference		0.052	Reference		0.048
	AG	1.912	1.054-3.468	0.033	2.088	1.109-3.932	0.023
	FOLFIRINOX	0.863	0.493-1.510	0.605	0.929	0.508-1.699	0.812
Tageted therapy	Absence	Reference		0.673	Reference		
	Presence	1.163	0.577-2.343				
HBsAg	Absence	Reference		0.865	Reference		
	Presence	0.916	0.332-2.524				
PD1	Absence	Reference		0.024	Reference		0.022
	Presence	0.563	0.342-0.927		0.537	0.316-0.913	

OS, overall survival; HR, hazard ratio; CI, confidence interval; NI, not include, other abbreviations as in **Table 1**.

### Comparisons of complications and adverse events between two groups

Complications were compared and no significant differences were identified between two groups in both of whole (**Supplementary Table S4**) and matched cohorts (**Table 4**). No treatment-related deaths occurred. In terms of surgery-related complications, similar probabilities of hemorrhage, pancreatic fistula, biliary fistula, abdominal infection, Pancreatitis, abscess, pain, cardiac arrhythmias, gastroparesis, and portal vein thrombosis were observed. Additionally, differences of incidences of immune-related adverse events, including loss of appetite, nausea, vomiting, and diarrhea were not statistically significant (all P>0.05).

**Table 4:** Comparisons of complications in matched cohorts.

Complication		Treatment		N	P	Complication		Treatment		N	P
		IRE	IRE+PD1					IRE	IRE+PD1		
hemorrhage	Absence	62	31	93	1.000	Diarrhea	Absence	60	32	92	0.298
	Presence	2	1	3			Presence	4	0	4	
Pancreatic fistula	Absence	54	29	83	0.534	Gastroparesis	Absence	62	32	94	0.551
	Presence	10	3	13			Presence	2	0	2	
Abdominal infection	Absence	59	32	91	0.166	Pancreatitis	Absence	63	32	95	0.667
	Presence	5	0	5			Presence	1	0	1	
Billional fistula	Absence	63	32	95	0.667	Abscess	Absence	62	31	93	1.000
	Presence	1	0	1			Presence	2	1	3	
Vomit	Absence	61	30	91	0.745	Pain	Absence	43	22	65	0.887
	Presence	3	2	5			Presence	21	10	31	
Loss of appetite	Absence	41	26	67	0.102	Arrhythmia	Absence	59	30	89	0.781
	Presence	23	6	29			Presence	5	2	7	
Nausea	Absence	60	32	92	0.298	Protal vein thrombosis	Absence	58	30	88	0.715
	Presence	4	0	4			Presence	6	2	8	

## Discussion

Minimally invasive local techniques such as endoscopic ultrasound-guided radiofrequency ablation (EUS-RFA) (26), high intensity focused ultrasound (HIFU) (27) and IRE have emerged as potential therapeutic options for pancreatic neoplastic lesions. Additionally, the feature of free from the heat sink effect makes IRE more appropriate in the treatment of LAPC. As a non-thermal ablation technique, irreversible IRE induces tumor cell apoptosis by irreversibly permeabilizing the cell membrane (8). Increasing clinical evidence supports the safety and efficacy of IRE in LAPC treatment (6, 25). Our previous studies further demonstrated that IRE combined with chemotherapy significantly improved patient survival compared to conventional treatments alone, including chemotherapy or conversion surgery (24, 28). These findings suggest that IRE plays a crucial role in LAPC management, and combining IRE with other modalities might further enhance therapeutic outcomes.

In recent years, breakthroughs in immunotherapies, particularly ICI and chimeric antigen receptor T-cell (CAR-T) therapies, have revolutionized cancer treatment (29, 30). However, the TME of PDAC often limits the effectiveness of immune therapies (31). Emerging studies, including our previous research, have shown that IRE not only destroys tumor cells but also modulates the local immune environment by promoting M1 macrophage polarization and increasing infiltration of tumor-specific T cells (9, 10, 19). This suggests that IRE could enhance immune responsiveness through increased antigen release and improved immune cell infiltration. Additionally, the low expression of anti-PD-1 therapy in PDAC may contribute to resistance against ICIs, but IRE-induced up-regulation of PD-1 expression on T cells might help overcome this barrier (32).

Building upon these observations, we hypothesized that IRE could enhance sensitivity to ICIs in LAPC, a concept supported by previous experimental studies demonstrating improved immunotherapy efficacy when combined with IRE (19). However, clinical validation of this combination has been lacking. To address this gap, we developed a novel treatment regimen involving IRE and chemotherapy followed by systemic administration of toripalimab, an anti-PD-1 antibody. In this study, we observed that patients receiving IRE combined with chemotherapy and toripalimab exhibited significantly improved immune profiles, tumor control, and survival compared with those undergoing IRE alone. Remarkably, median overall survival in the combination group reached near three years, highlighting its potential as an effective therapeutic strategy for LAPC.

The enhanced immune activity could contribute to the significantly elevated efficacy of combination therapy. In our previous studies, It was found that notable increases in circulating CD4^+^ helper T cells and CD8^+^ cytotoxic T cells, alongside decreases in immunosuppressive CD8^+^ regulatory T cells following combined therapy. Furthermore, significant elevations of cytokines such as IL-4, IL-6, IL-10, TNF, and IFN-γ were observed, reflecting a robust antitumor immune response. Elevated TNF and IFN-γ, primarily secreted by activated CD8^+^ T cells, indicate enhanced specific immune-mediated tumor killing. TNF-α also promotes M1 macrophage polarization, facilitating antigen presentation and immune activation via additional cytokines such as IL-4, IL-6, and IL-10 (21). These immunological changes likely underpin the improved survival outcomes observed with combined treatment.

Our study confirmed the synergistic benefit of adding anti-PD-1 therapy to IRE in LAPC management without significantly increasing adverse events, corroborating previous safety data (21, 33). Administering toripalimab one week after IRE treatment provided an optimal time window for immune activation and patient recovery, potentially contributing to the low incidence of adverse events.

Despite promising results, our study has several limitations. Firstly, its retrospective, non-randomized design may introduce selection bias, despite the balanced baseline characteristics and the omission of important indices, such as Quality of Life (QoL) assessments. Prospective, randomized controlled trials are necessary to validate our findings. Secondly, study cohort based on single center limits generalizability, emphasizing the need for validation in populations from multiple centers. Further randomized clinical trials with longer follow-up periods are required to confirm the enduring efficacy of this novel combination therapy.

## Data Availability

The raw data supporting the conclusions of this article will be made available by the authors, without undue reservation.
