# Supplementary material for: Efficacy of irreversible electroporation combined with immunotherapy versus irreversible electroporation alone in locally advanced pancreatic cancer: a propensity score-matched retrospective study
Source: Front Immunol. 2025 Jul 8;16:1620988. doi: 10.3389/fimmu.2025.1620988 (PMC12279750; doi:10.3389/fimmu.2025.1620988)
Supplement: Supplementary file 1 [file Table1.docx]

Supplementary Table 1. Clinicopathological characteristics of patients in matched cohort.

| Variable | | Treatment | | N | P | Variable | | Treatment | | N | P |
| --- | --- | --- | --- | --- | --- | --- | --- | --- | --- | --- | --- |
|  |  | IRE | IRE+PD1 | 96 |  |  |  | IRE | IRE+PD1 | 96 |  |
| Age (years) | ≤ 60 | 39 | 18 | 57 | 0.666 | Tumor grade | Well | 6 | 2 | 8 | 0.583 |
|  | > 60 | 25 | 14 | 39 |  |  | Moderate | 35 | 21 | 56 |  |
| Gender | Male | 33 | 11 | 44 | 0.132 |  | Poor | 23 | 9 | 32 |  |
|  | Female | 31 | 21 | 52 |  | Tumor size (cm) | ≤ 2 | 1 | 1 | 2 | 0.669 |
| WBC (*10^9^) | ≤ 10 | 58 | 30 | 88 | 0.715 |  | 2~4 | 42 | 23 | 65 |  |
|  | > 10 | 6 | 2 | 8 |  |  | > 4 | 21 | 8 | 29 |  |
| HGB (g/L) | ≤ 125 | 25 | 8 | 33 | 0.254 | Tumor site | Head | 31 | 14 | 45 | 0.828 |
|  | > 125 | 39 | 24 | 63 |  |  | Body/tail | 33 | 18 | 51 |  |
| PLT (*10^9^) | ≤ 350 | 60 | 27 | 87 | 0.155 | Imaging LN metastasis | Absence | 30 | 10 | 40 | 0.189 |
|  | > 350 | 4 | 5 | 9 |  |  | Presence | 34 | 22 | 56 |  |
| ALT (U/L) | ≤ 50 | 56 | 26 | 82 | 0.541 | Vascular invasion type | Vein | 54 | 29 | 83 | 0.534 |
|  | > 50 | 8 | 6 | 14 |  |  | Artery | 10 | 3 | 13 |  |
| AST (U/L) | ≤ 40 | 55 | 30 | 85 | 0.327 | Response to NAC | PR | 19 | 15 | 34 | 0.252 |
|  | > 40 | 9 | 2 | 11 |  |  | SD | 37 | 14 | 51 |  |
| ALP (U/L) | ≤ 125 | 38 | 20 | 58 | 0.827 |  | PD | 8 | 3 | 11 |  |
|  | > 125 | 26 | 12 | 38 |  | Tageted therapy | Absence | 59 | 26 | 85 | 0.172 |
| GGT (U/L) | ≤ 60 | 36 | 23 | 59 | 0.183 |  | Presence | 5 | 6 | 11 |  |
|  | > 60 | 28 | 9 | 37 |  | HBsAg | Absence | 62 | 30 | 92 | 0.599 |
| ALB (g/L) | > 40 | 12 | 6 | 18 | 1.000 |  | Presence | 2 | 2 | 4 |  |
|  | ≤ 40 | 52 | 26 | 78 |  | Adjuvant chemotherapy | S-1 | 41 | 18 | 59 | 0.740 |
| TBIL (umol/L) | ≤ 20.5 | 51 | 27 | 78 | 0.782 |  | AG | 9 | 6 | 15 |  |
|  | > 20.5 | 13 | 5 | 18 |  |  | FOLFIRINOX | 14 | 8 | 22 |  |
| IBIL (umol/L) | ≤ 15 | 56 | 30 | 86 | 0.488 | Radiotherapy | Absence | 50 | 22 | 72 | 0.329 |
|  | > 15 | 8 | 2 | 10 |  |  | Presence | 14 | 10 | 24 |  |
| CRP (ng/L) | ≤3 | 45 | 22 | 67 | 0.875 | CA19-9 (U/ml) | ≤ 35 | 20 | 12 | 32 | 0.647 |
|  | > 3 | 19 | 10 | 29 |  |  | >35 | 44 | 20 | 64 |  |
| CEA (ng/ml) | ≤ 5 | 41 | 26 | 67 | 0.102 | CA125 | ≤ 35 | 49 | 30 | 79 | 0.072 |
|  | > 5 | 23 | 6 | 29 |  |  | >35 | 15 | 2 | 17 |  |

Abbreviations: WBC, white blood cell; PLT, platelet; ALT, alanine transaminase; AST, aspartate aminotransferase; ALP, alkaline phosphatase; GGT, glutamyl transpeptidase; ALB, albumin; TBIL, total bilirubin; IBIL, indirect bilirubin; CRP, C-reactive protein; HBsAg, hepatitis B surface antigen; CEA, carcinoembryonic antigen; CA19-9, carbohydrate antigen 19-9; AG, Abraxane-GEM; FOLFIRINOX, leucovorin, fluorouracil, irinotecan, and oxalipatin; LN, lymph node; NAC, neoadjuvant chemotherapy.

Supplementary Table 2. Independent prognostic factors for OS.

| Characteristics | | OS | | | | | |
| --- | --- | --- | --- | --- | --- | --- | --- |
|  |  | Univariate analysis | | | Multivariate analysis | | |
|  |  | HR | 95%CI | P | HR | 95% | P |
| Age (years) | ≤ 60 | reference |  | 0.025 | Reference |  | 0.031 |
|  | > 60 | 1.746 | 1.072-2.844 |  | 1.816 | 1.056-3.124 |  |
| Gender | Male | reference |  | 0.187 | Reference |  |  |
|  | Female | 0.719 | 0.441-1.173 |  |  |  |  |
| WBC (*10^9^) | ≤ 10 | reference |  | 0.777 | Reference |  |  |
|  | > 10 | 0.885 | 0.381-2.056 |  |  |  |  |
| HGB (g/L) | ≤ 120 | reference |  | 0.330 | Reference |  |  |
|  | > 120 | 0.772 | 0.458-1.300 |  |  |  |  |
| PLT (*10^9^) | ≤ 300 | reference |  | 0.938 | Reference |  |  |
|  | > 300 | 1.030 | 0.490-2.163 |  |  |  |  |
| ALT (U/L) | ≤ 50 | reference |  | 0.427 | Reference |  |  |
|  | > 50 | 1.272 | 0.702-2.307 |  |  |  |  |
| AST (U/L) | ≤ 40 | reference |  | 0.074 | Reference |  | 0.280 |
|  | > 40 | 1.786 | 0.945-3.375 |  | 1.458 | 0.735-2.892 |  |
| ALP (U/L) | ≤ 125 | Reference |  | 0.290 | Reference |  |  |
|  | > 125 | 0.763 | 0.462-1.260 |  |  |  |  |
| GGT (U/L) | ≤ 60 | Reference |  | 0.751 | Reference |  |  |
|  | > 60 | 1.085 | 0.657-1.791 |  |  |  |  |
| ALB (g/L) | > 40 | Reference |  | 0.109 | Reference |  |  |
|  | ≤ 40 | 0.629 | 0.356-1.109 |  |  |  |  |
| TBIL (umol/L) | ≤ 20.5 | Reference |  | 0.685 | Reference |  |  |
|  | > 20.5 | 0.884 | 0.488-1.601 |  |  |  |  |
| IBIL(umol/L) | ≤ 15 | Reference |  | 0.996 | Reference |  |  |
|  | > 15 | 1.002 | 0.477-2.104 |  |  |  |  |
| CRP (ng/L) | ≤3 | Reference |  | 0.110 | Reference |  |  |
|  | > 3 | 1.513 | 0.910-2.515 |  |  |  |  |
| CEA (ng/mL) | ≤ 5 | Reference |  | 0.501 | Reference |  |  |
|  | > 5 | 0.829 | 0.480-1.432 |  |  |  |  |
| CA19-9 (U/ml) | ≤ 35 | Reference |  | 0.165 | Reference |  |  |
|  | >35 | 1.471 | 0.853-2.538 |  |  |  |  |
| CA125 | ≤ 35 | Reference |  | 0.627 | Reference |  |  |
|  | >35 | 1.160 | 0.637-2.112 |  |  |  |  |
| Tumor size | ≤ 2 | Reference |  | 0.145 | Reference |  |  |
|  | 2~4 | 2.808 | 0.381-20.618 | 0.311 |  |  |  |
|  | > 4 | 4.333 | 0.568-33.029 | 0.157 |  |  |  |
| Tumor site | Head | Reference |  | 0.266 | Reference |  |  |
|  | Body/tail | 0.755 | 0.460-1.239 |  |  |  |  |
| Tumor grade | Well | Reference |  | 0.089 | Reference |  | 0.029 |
|  | Moderate | 1.577 | 0.484-5.142 | 0.450 | 3.010 | 0.843-10.751 | 0.090 |
|  | Poor | 2.583 | 0.779-8.571 | 1.121 | 4.735 | 1.312-17.088 | 0.018 |
| Imaging LN metastasis | Absence | Reference |  | 0.259 | Reference |  |  |
|  | Presence | 0.750 | 0.456-1.236 |  |  |  |  |
| Vascular invasion type | Vein | Reference |  | 0.040 | Reference |  | 0.017 |
|  | Artery | 1.939 | 1.031-3.647 |  | 2.324 | 1.166-4.633 |  |
| Neoadjuvant radiotherapy | Absence | Reference |  | 0.163 | Reference |  |  |
|  | Presence | 0.645 | 0.348-1.194 |  |  |  |  |
| Response to NCP | PR | Reference |  | 0.028 | Reference |  | 0.181 |
|  | SD | 2.154 | 1.196-3.877 | 0.011 | 1.607 | 0.871-2.963 | 0.129 |
|  | PD | 2.583 | 0.779-8.571 | 1.121 | 0.905 | 0.337-2.432 | 0.843 |
| Adjuvant chemotherapy | S-1 | Reference |  | 0.200 | Reference |  |  |
|  | AG | 1.215 | 0.607-2.434 | 0.582 |  |  |  |
|  | FOLFIRINOX | 0.598 | 0.314-1.139 | 0.118 |  |  |  |
| Tageted therapy | Absence | Reference |  | 0.571 | Reference |  |  |
|  | Presence | 0.783 | 0.337-1.823 |  |  |  |  |
| HBsAg | Absence | Reference |  | 0.395 | Reference |  |  |
|  | Presence | 0.604 | 0.189-1.930 |  |  |  |  |
| PD1 | Absence |  |  | 0.010 | Reference |  | 0.025 |
|  | Presence | 0.466 | 0.261-0.832 |  | 0.497 | 0.269-0.917 |  |

Abbreviations: OS, overall survival; HR, hazard ratio; CI, confidence interval; NI, not include, other abbreviations as in Table 1.

Supplementary Table 3. Independent prognostic factors for PFS.

| Characteristics | | OS | | | | | |
| --- | --- | --- | --- | --- | --- | --- | --- |
|  |  | Univariate analysis | | | Multivariate analysis | | |
|  |  | HR | 95%CI | P | HR | 95% | P |
| Age (years) | ≤ 60 | reference |  | 0.353 | Reference |  |  |
|  | > 60 | 1.224 | 0.799-1.874 |  |  |  |  |
| Gender | Male | reference |  | 0.303 | Reference |  |  |
|  | Female | 1.253 | 0.816-1.922 |  |  |  |  |
| WBC (*10^9^) | ≤ 10 | reference |  | 0.309 | Reference |  |  |
|  | > 10 | 0.668 | 0.308-1.453 |  |  |  |  |
| HGB (g/L) | ≤ 120 | reference |  | 0.798 | Reference |  |  |
|  | > 120 | 1.063 | 0.667-1.694 |  |  |  |  |
| PLT (*10^9^) | ≤ 300 | reference |  | 0.339 | Reference |  |  |
|  | > 300 | 0.700 | 0.337-1.455 |  |  |  |  |
| ALT (U/L) | ≤ 50 | reference |  | 0.646 | Reference |  |  |
|  | > 50 | 0.880 | 0.509-1.520 |  |  |  |  |
| AST (U/L) | ≤ 40 | reference |  | 0.689 | Reference |  |  |
|  | > 40 | 1.129 | 0.622-2.050 |  |  |  |  |
| ALP (U/L) | ≤ 125 | Reference |  | 0.110 | Reference |  |  |
|  | > 125 | 0.699 | 0.451-1.085 |  |  |  |  |
| GGT (U/L) | ≤ 60 | Reference |  | 0.967 | Reference |  |  |
|  | > 60 | 1.009 | 0.652-1.562 |  |  |  |  |
| ALB (g/L) | > 40 | Reference |  | 0.623 | Reference |  |  |
|  | ≤ 40 | 0.877 | 0.520-1.479 |  |  |  |  |
| TBIL (umol/L) | ≤ 20.5 | Reference |  | 0.292 | Reference |  |  |
|  | > 20.5 | 0.751 | 0.440-1.280 |  |  |  |  |
| IBIL(umol/L) | ≤ 15 | Reference |  | 0.493 | Reference |  |  |
|  | > 15 | 1.261 | 0.650-2.444 |  |  |  |  |
| CRP (ng/L) | ≤3 | Reference |  | 0.004 | Reference |  | 0.142 |
|  | > 3 | 1.924 | 1.230-3.011 |  | 1.475 | 0.878-2.478 |  |
| CEA (ng/mL) | ≤ 5 | Reference |  | 0.983 | Reference |  |  |
|  | > 5 | 1.005 | 0.635-1.589 |  |  |  |  |
| CA19-9 (U/ml) | ≤ 35 | Reference |  | 0.055 | Reference |  | 0.470 |
|  | >35 | 1.589 | 0.990-2.551 |  | 1.213 | 0.719-2.046 |  |
| CA125 | ≤ 35 | Reference |  | 0.838 | Reference |  |  |
|  | >35 | 1.058 | 0.617-1.815 |  |  |  |  |
| Tumor size | ≤ 2 | Reference |  | 0.059 | Reference |  | 0.302 |
|  | 2~4 | 3.859 | 0.527-28.264 | 0.184 | 3.452 | 0.446-26.695 | 0.235 |
|  | > 4 | 5.986 | 0.797-44.937 | 0.082 | 4.438 | 0.543-36.283 | 0.165 |
| Tumor site | Head | Reference |  | 0.915 | Reference |  |  |
|  | Body/tail | 0.977 | 0.633-1.507 |  |  |  |  |
| Tumor grade | Well | Reference |  | 0.710 | Reference |  |  |
|  | Moderate | 0.912 | 0.413-2.016 | 0.820 |  |  |  |
|  | Poor | 1.109 | 0.483-2.545 | 0.807 |  |  |  |
| Imaging LN metastasis | Absence | Reference |  | 0.053 | Reference |  | 0.111 |
|  | Presence | 0.650 | 0.420-1.006 |  | 0.683 | 0.428-1.091 |  |
| Vascular invasion type | Vein | Reference |  | 0.393 | Reference |  |  |
|  | Artery | 1.296 | 0.715-2.350 |  |  |  |  |
| Neoadjuvant radiotherapy | Absence | Reference |  | 0.969 | Reference |  |  |
|  | Presence | 0.990 | 0.605-1.622 |  |  |  |  |
| Response to NCP | PR | Reference |  |  | Reference |  |  |
|  | SD | 1.234 | 0.770-1.977 | 0.383 |  |  |  |
|  | PD | 0.886 | 0.400-1.960 | 0.764 |  |  |  |
| Adjuvant chemotherapy | S-1 | Reference |  | 0.039 | Reference |  | 0.039 |
|  | AG | 1.897 | 1.066-3.378 | 0.029 | 2.216 | 1.190-4.129 | 0.012 |
|  | FOLFIRINOX | 0.804 | 0.468-1.381 | 0.430 | 1.066 | 0.584-1.946 | 0.834 |
| Tageted therapy | Absence | Reference |  | 0.689 | Reference |  |  |
|  | Presence | 1.152 | 0.576-2.307 |  |  |  |  |
| HBsAg | Absence | Reference |  | 0.908 | Reference |  |  |
|  | Presence | 0.952 | 0.412-2.197 |  |  |  |  |
| PD1 | Absence |  |  | 0.026 | Reference |  | 0.020 |
|  | Presence | 0.580 | 0.359-0.936 |  | 0.537 | 0.318-0.909 |  |

Abbreviations: OS, overall survival; HR, hazard ratio; CI, confidence interval; NI, not include, other abbreviations as in Table 1.

Supplementary Table 4. Comparisons of complications in two treatment groups.

| Complication | | Treatment | | N | P | Complication | | Treatment | | N | P |
| --- | --- | --- | --- | --- | --- | --- | --- | --- | --- | --- | --- |
|  |  | IRE | IRE+PD1 |  |  |  |  | IRE | IRE+PD1 |  |  |
| hemorrhage | Absence | 74 | 31 | 105 | 0.887 | Diarrhea | Absence | 72 | 32 | 104 | 0.316 |
|  | Presence | 2 | 2 | 3 |  |  | Presence | 4 | 0 | 4 |  |
| Pancreatic fistula | Absence | 66 | 29 | 95 | 0.751 | Gastroparesis | Absence | 74 | 32 | 106 | 0.885 |
|  | Presence | 10 | 3 | 13 |  |  | Presence | 2 | 0 | 2 |  |
| Abdominal infection | Absence | 71 | 32 | 103 | 0.319 | Pancreatitis | Absence | 75 | 32 | 107 | 0.514 |
|  | Presence | 5 | 0 | 5 |  |  | Presence | 1 | 0 | 1 |  |
| Billional fistula | Absence | 75 | 32 | 107 | 0.704 | Abscess | Absence | 74 | 31 | 105 | 0.887 |
|  | Presence | 1 | 0 | 1 |  |  | Presence | 2 | 1 | 3 |  |
| Vomit | Absence | 73 | 30 | 103 | 0.632 | Pain | Absence | 54 | 22 | 76 | 0.821 |
|  | Presence | 3 | 2 | 5 |  |  | Presence | 22 | 10 | 32 |  |
| Loss of appetitle | Absence | 53 | 26 | 79 | 0.245 | Arrhythmia | Absence | 71 | 30 | 101 | 0.949 |
|  | Presence | 23 | 6 | 29 |  |  | Presence | 5 | 2 | 7 |  |
| Nausea | Absence | 72 | 32 | 104 | 0.316 | Protal vein thrombosis | Absence | 70 | 30 | 100 | 0.766 |
|  | Presence | 4 | 0 | 4 |  |  | Presence | 6 | 2 | 8 |  |
